# The Galapagos giant tortoise *Chelonoidis phantasticus* is not extinct

**DOI:** 10.1038/s42003-022-03483-w

**Published:** 2022-06-09

**Authors:** Evelyn L. Jensen, Stephen J. Gaughran, Nicole A. Fusco, Nikos Poulakakis, Washington Tapia, Christian Sevilla, Jeffreys Málaga, Carol Mariani, James P. Gibbs, Adalgisa Caccone

**Affiliations:** 1 School of Natural and Environmental Sciences, Newcastle University, Newcastle Upon Tyne, UK; 2 Department of Ecology & Evolutionary Biology, Princeton University, Princeton, NJ USA; 3 Department of Ecology and Evolutionary Biology, Yale University, New Haven, CT USA; 4 Department of Biology, School of Sciences and Engineering, University of Crete, Irakleio, Greece; 5 The Natural History Museum of Crete, School of Sciences and Engineering, University of Crete, Heraklion, Greece; 6 Institute of Molecular Biology and Biotechnology, Foundation for Research and Technology-Hellas, Heraklion, Greece; 7 Galapagos Conservancy, Fairfax, VA USA; 8 University of Málaga, Campus Teatinos, Apdo, 59.29080 Málaga, Spain; 9 Conservation and Restoration of Insular Ecosystems Department, Galapagos National Park Directorate, Puerto Ayora, Galapagos Ecuador; 10 Department of Molecular, Cellular and Developmental Biology, Yale University, New Haven, CT USA; 11 Department of Environmental Biology, College of Environmental Science and Forestry, State University of New York, Syracuse, NY USA

**Keywords:** Phylogenetics, Population genetics

## Abstract

The status of the Fernandina Island Galapagos giant tortoise (*Chelonoidis phantasticus*) has been a mystery, with the species known from a single specimen collected in 1906. The discovery in 2019 of a female tortoise living on the island provided the opportunity to determine if the species lives on. By sequencing the genomes of both individuals and comparing them to all living species of Galapagos giant tortoises, here we show that the two known Fernandina tortoises are from the same lineage and distinct from all others. The whole genome phylogeny groups the Fernandina individuals within a monophyletic group containing all species with a saddleback carapace morphology and one semi-saddleback species. This grouping of the saddleback species is contrary to mitochondrial DNA phylogenies, which place the saddleback species across several clades. These results imply the continued existence of lineage long considered extinct, with a current known population size of a single individual.

## Abstract (web summary)

Based on genomic data, the Galapagos giant tortoise species native to Fernandina Island appears to be alive and well, survived by at least one female after being considered extinct since 1906.

## Introduction

Since 1906, evidence has accrued that a mysterious species of giant tortoise might exist on Fernandina Island, an active volcano that stands alone on western periphery of the Galapagos Archipelago and is reputed to be the largest pristine island on Earth. A single specimen of *Chelonoidis phantasticus*—“The Fantastic Giant Tortoise”—was collected by the explorer Rollo Beck during an expedition by the California Academy of Sciences in 1906^1^. The fantastic nature of the Fernandina giant tortoise is due to the extraordinary morphology of the male specimen, with extreme flaring of its marginal scutes and unusually prominent “saddlebacking” of the front section of the carapace, unlike any other tortoise yet observed in Galapagos, or elsewhere on the planet as saddlebacking is unique to Galapagos tortoises^2^. Despite being known previously from only one specimen, the Fernandina tortoise has been considered to represent a distinct taxon: a previous analysis of 1682 base pairs of mitochondrial DNA obtained from this specimen showed 1.8% sequence divergence from the other Galapagos tortoise species, and produced a phylogeny in which the Fernandina Island tortoise is sister to one of the Santa Cruz Island species, *C. porteri*^3^.

**Fig. 1:**
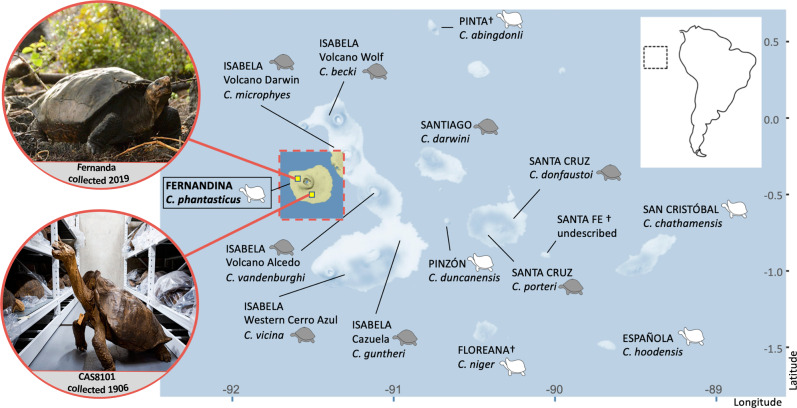
Map of the Galapagos Archipelago, indicating the approximate locations on Fernandina Island where the *Chelonoidis phantasticus* individuals were found in 1906 and 2019. Island names are in capital letters, species names are in italics. Tortoise icons indicate the morphology of the species, either domed (gray), saddleback (white), or semi-saddleback (indicated with both icons present). Map tiles by Stamen Design, under CC BY 3.0. Data by OpenStreetMap, under ODbL. Image of Fernanda by Lucas Bustamante © Galapagos Conservancy, image of the historical specimen Kathryn Whitney © California Academy of Sciences.

The radiation of giant tortoises endemic to the Galapagos consists of 14 named taxa (Fig. 1), all of which evolved from a single ancestor that colonized the volcanic archipelago within the last 3 million years^3–6^. Due to the recent nature of the radiation, there is debate as to whether the taxa should be considered species or subspecies, since their diversification is younger than almost any other congeneric pair of tortoise species^7,8^. However, a criterion of minimum clade age does not correspond to any of the commonly recognized species concepts^9^. Therefore, we follow the taxonomy of Rhodin, et al.^10^ in recognizing 14 named species. The balance of evidence overwhelmingly indicates that each lineage is highly distinct based on thousands of genome-wide markers^11–13^, demonstrating that they are separately evolving as lineages within a larger metapopulation, which is the core concept within all species concepts^9^.

Diversification of Galapagos tortoises has followed the island progression rule, with the oldest islands being home to the earliest diverging lineages^6^. There is a continuum of morphological variation in carapace shape that is genetically based, with morphology linked to the environmental conditions in the species habitat. On one end of the continuum are species with a domed carapace, which typically live in more mesic, higher elevation ecosystems, whereas those with the saddleback form inhabit more arid, lower elevation environments^14^. Two species, *C. darwini* and *C. chathamensis*, exhibit the midpoint of this continuum, with morphology described as “semi-saddleback”. All species are listed on the IUCN Red List as either Vulnerable, Endangered, Critically Endangered, or Extinct^15^, their populations having been decimated during the 19–20th centuries through exploitation by humans, and the negative impacts of invasive species^16^.

Whether the Fernandina tortoise lives on has intrigued biologists for over a century. The island has remained largely unexplored due to extensive lava fields barring access to much of the island’s interior, resulting in little exploration beyond the island’s coastline. Nevertheless, in the century since Beck’s discovery, anecdotal evidence of tortoises on the island has accrued. Eighteen scats attributable to tortoises were reported on the western slopes of the island in 1964^17^, scats and a possible visual observation from an aircraft were reported during the early 2000’s, and another possible tortoise scat was seen in 2014 (J. Málaga, personal observation).

Finally, in 2019 a surprise discovery triggered a wave of international news that the giant tortoises may yet survive on Fernandina Island. A single female tortoise was found on the lower, northwestern flank of the volcano (Fig. 1). The tortoise nicknamed “Fernanda” was found in an isolated patch of xerophytic vegetation, cut off from the main vegetated area on the southeast of the island by several lava flows. She is likely well over 50 years old but is small with her growth stunted. Fernanda is now in captivity in the Galapagos National Park Tortoise Center. Encouragingly, recent signs (i.e., tracks, scat) of at least 2–3 other tortoises were found during other expeditions on the island (W. Tapia, J. Málaga, personal observations).

Despite the intense interest in the rediscovery of this purportedly lost species, Fernanda’s status as a *phantasticus* tortoise has remained in question. She lacks the striking saddleback flaring of the male historical specimen (Fig. 1); however, her stunted growth may have distorted her morphological features, rendering conclusions based on morphology tenuous. Additionally, there is a history of mariners moving tortoises between islands in historical times, which has resulted in groups of tortoises with mixed ancestry on the nearby island Isabela^18–21^, making it plausible that Fernanda could be a transplant from another island.

To investigate the relationship between the two tortoises found on Fernandina Island more than 100 years apart, we sequenced the full genomes of the historical specimen collected in 1906 (“CAS 8101”) and Fernanda, and compared them to a dataset of genomes of three individuals from each of the 12 living lineages^12^, and one individual of the extinct *C. abingdonii*^22^.

## Results

**Fig. 2:**
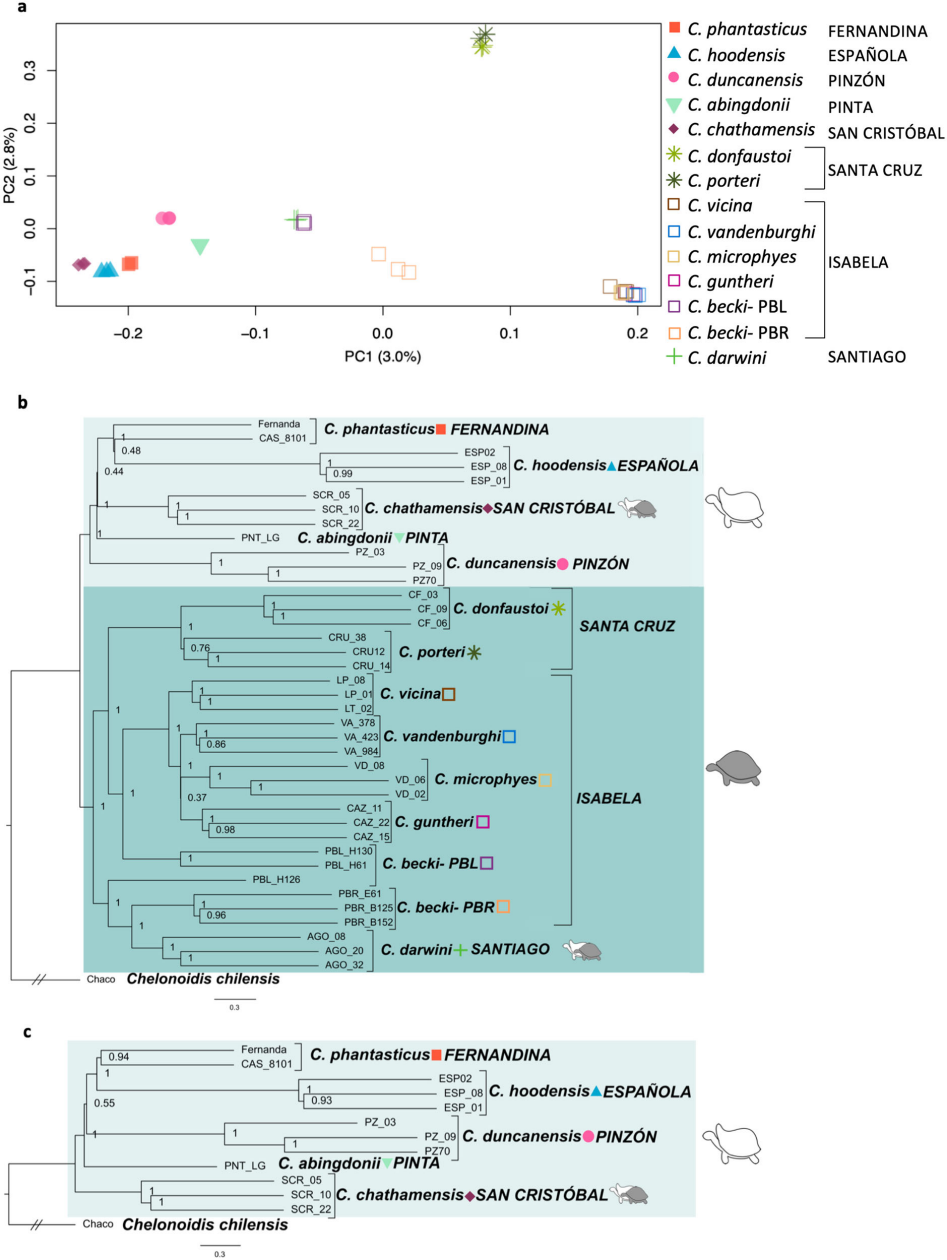
Genetic relationships among Galapagos giant tortoise species, showing the affinity between the two tortoises yet found on Fernandina Island. **a** Plot of the first two principal components axes. Percentages on the axes correspond to the amount of variation explained by that axis. **b** Astral consensus tree using 14 species, created from 2331 maximum likelihood RAxML gene trees. The lighter box highlights the clade with predominantly saddleback carapace morphology, the darker box indicates the clade with predominantly domed morphology, the exceptions being the semi-saddlebacked species which are indicated by having both icons next to them. **c** Astral consensus tree using the predominantly saddlebacked species, created from 2099 maximum likelihood trees. For both B and C, 100 kb segments of the genome were used for the gene trees, each spaced 100 kb apart. Species names are in italics, island names are in capital letters. Values on the nodes indicate posterior probabilities.

To evaluate the similarity between the two Fernandina specimens’ genomes (CAS 8101 with 22× coverage, Fernanda at 34× coverage, see supplementary text for more details) and how different they are from other Galapagos tortoises, we conducted a Principal Components Analysis (PCA) based on >750,000 SNPs. This analysis clearly clusters the two Fernandina individuals together and distinct from individuals belonging to all other species (Fig. 2A).

To investigate the phylogenetic position of the two Fernandina Island tortoises we carried out phylogenomic analyses on the same nuclear genomic dataset. As sequence segment length and linkage disequilibrium can impact tree topology, we created four datasets of different sized genomic segments (10 and 100 kb) spaced at different distances (100 kb and 1 Mb). For each dataset, we built maximum likelihood gene trees from each segment using RAxML^23^ and generated a final species tree using ASTRAL^24^, a method that infers true species trees under the multi-species coalescent model. All datasets had strong support for two main clades of Galapagos giant tortoises: one including all saddleback tortoises and the semi-saddleback species *C. chathamensis*, and the other including all the domed tortoises and the semi-saddleback species *C. darwini* (Fig. 2B and Figs. S1–3). However, because the placement of the Fernandina tortoises was not well resolved, we repeated the analysis focusing only on the clade of predominantly saddleback tortoises, including the two Fernandina tortoises and all ten individuals from the three saddleback species from the islands Pinzón, Española, and Pinta, and the semi-saddleback species from San Cristóbal. The phylogenies of this subset of tortoises show strong support for each of the known species, including a strongly supported monophyletic clade comprised of the two Fernandina tortoises (Fig. 2C and Figs. S4–6).

**Fig. 3:**
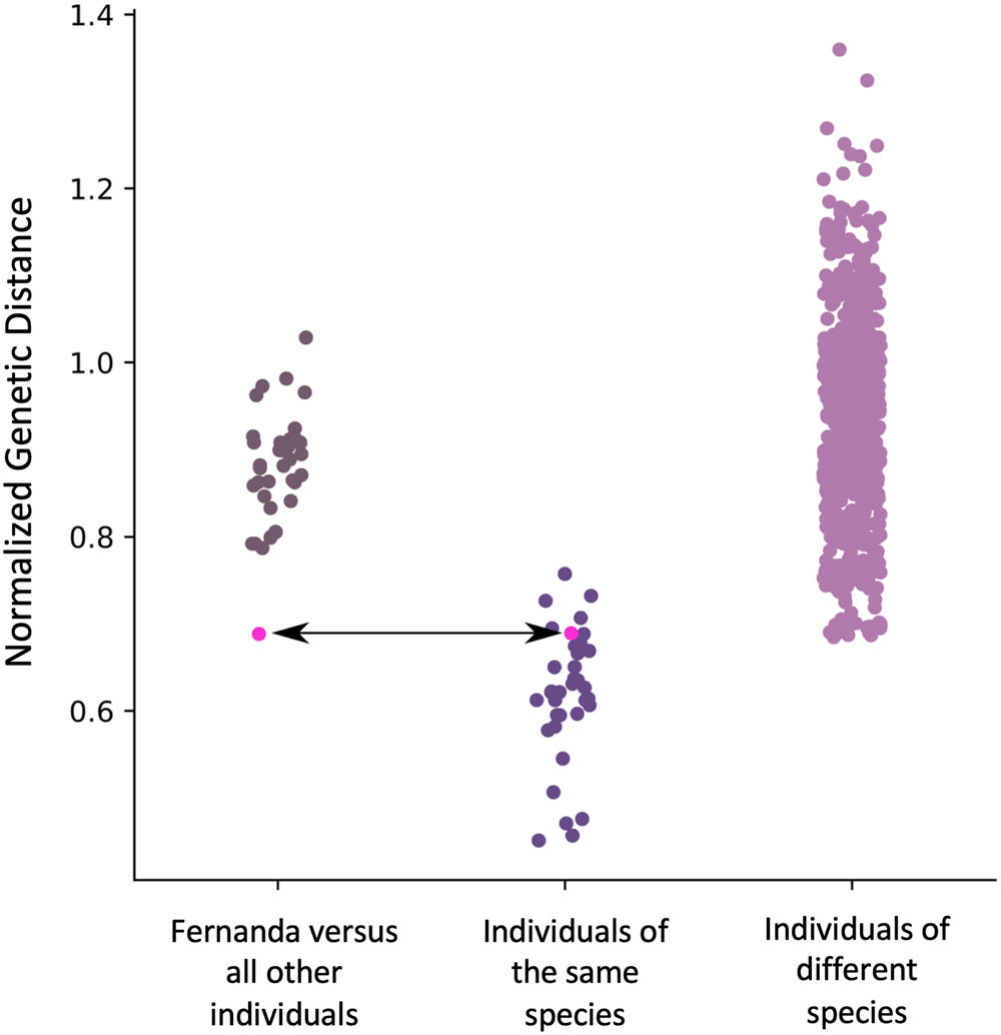
Genetic distance between pairs of individuals at different hierarchical levels. Distances between Fernanda and each other tortoise, between individuals of the same species, and between individuals of different species. The comparisons of Fernanda and CAS 8101 are highlighted in magenta with the arrow pointing to them. All distances are calculated from the dataset of 100 kb segments separated by 100 kb.

To quantify the genomic patterns reflected in the phylogenies and the PCA, we measured corrected genetic distances between pairs of genomes (Eq. 1 in the “Methods”) using the genomic dataset from our main phylogenies (i.e., 100 kb long segments separated by 100 kb). When averaged across the genome, genetic distances are smaller between individuals of the same species than between individuals of different species. We show that the genetic distance between the two tortoises from Fernandina is smaller than the distance between Fernanda and any of the other tortoises sequenced, and that this distance is similar to the intraspecific differences between individuals of the same species (Fig. 3). Taken together, these analyses indicate that the tortoise found on Fernandina Island in 2019 belongs to the same genetic lineage as the historical *C. phantasticus* specimen, and that these two individuals are genetically distinct from other species of giant tortoise found on other islands in the Galapagos archipelago.

## Discussion

Genome-wide sequencing of the only two giant tortoises ever found on Fernandina Island reveal they are from the same lineage and distinct from all others. The population genomic and phylogenomic analyses performed in this study corroborate previous findings about the evolutionary history of the Galapagos giant tortoise species, while also elucidating the complicated nature of studying recent island radiations. With our analyses, we show that even a whole-genome approach using millions of base pairs of sequence has methodological limitations in building bifurcating phylogenies of recently diverged species radiations. Most notably, as discussed above, the Fernandina clade (CAS 8101 and Fernanda) has weak or ambiguous support when the phylogeny is built from the genomic sequences of all 39 Galapagos giant tortoise individuals (Figs. S1–3). This runs contrary to the close genetic relationship between CAS 8101 and Fernanda observed in the phylogenies using the subclade and the PCA, and suggests that the increasing number of possible trees with additional taxa overwhelms the ability to discriminate between closely related species when incomplete lineage sorting (ILS) or historical gene flow may be common. The fact that including only tortoises belonging to the subclade provides better resolution for each species’ clade suggests that ILS and extremely short coalescence times create phylogenetic noise in a significant proportion of the gene trees containing many individuals. Furthermore, here we use unphased genomic data, with the pseudo-random selection of alleles based on allelic read count. This strategy may further exacerbate the problems of ILS, historical gene flow, and recent hybridization by drawing together lineages that are phylogenetically divergent. Current phasing methods (e.g., the generation of haplotype reference panels^25^ or through long-read sequencing^26^) are still expensive or impossible using short-read sequencing data. Future alternatives to this strategy driven by advances in sequencing technology and in statistical genomics, including the use of phased haplotypes^27^ and spatially explicit genome-wide genealogies^28^, may help to better resolve the evolutionary history of these organisms by building robust genealogies and phylogenies of haplotypes within each species.

While the analyses based on the nuclear genome group the two Fernandina specimens together and sister to *C. hoodensis*, phylogenetic analyses using only the mitochondrial genome place the Fernandina mitogenomes in different clades (Fig. S7). In the mitochondrial phylogeny, CAS 8101 is sister to *C. porteri* from Santa Cruz Island (consistent with previous studies^3^), whereas Fernanda is sister to the extinct species, *C. niger*, from Floreana Island. Unfortunately, we have not been able to obtain a nuclear genome sequence for the extinct *C. niger* to further explore this relationship. There is evidence of *C. niger* individuals being introduced by humans to the *C. becki* population nearby on Isabela Island^19,20^, something that might also have transpired on Fernandina Island. In addition to the discordant position of the two Fernandina tortoises in the mitochondrial and nuclear trees, there are other topological discordances between the mtDNA and the nuclear trees for some other species (see Supplementary text). Understanding the evolutionary history underlying this discordance will require further study, ideally using representatives from all of the species, including the extinct ones, as incomplete taxon sampling can lead to errors in the estimation of topology^29^, and is likely to leave gaps in our understanding of the phylogeography of the group.

Levels of genome wide diversity can provide insights on a species demographic history and on the likelihood of species recovery if other individuals are found. Although rare species reduced to only a few individuals typically display low genomic diversity as consequence of multiple small population phenomena^30^, this does not seem to be the case for the two Fernandina specimens. Despite being the only known individuals of their species, CAS 8101 and Fernanda show very high genome-wide heterozygosity compared to individuals of other Galapagos giant tortoise species (Table S1). This pattern of high heterozygosity is consistent across estimator methods, when sequencing depth is downsampled, and when only transversions are used to account for possible DNA damage (Supplementary Data 1). It is unclear how the history of the Fernandina tortoise has led them to have such high diversity, although historical gene flow/hybridization or a large historical population size are plausible explanations. Whatever the case, the strikingly high genetic diversity observed in Fernanda bodes well for species recovery should other Fernandina tortoises yet be found.

Fernanda can be identified with confidence as a *C. phantasticus* tortoise, yet she represents just a single living individual. If any additional surviving tortoises are found on Fernandina Island, they are unlikely to number more than a few individuals given that they must have evaded detection during previous extensive searches of the island. Fernandina Island is the most active volcano in the Galapagos, with more than 25 eruptions in the past two centuries^31^, which likely poses a direct threat to the persistence of a tortoise population there. Vulcanism has generated the current habitat mosaic for these tortoises: small and fragmented patches of marginal vegetation, with little access to suitable nesting areas. Removing to captivity any other individuals discovered on the island may be the best strategy to ensure their safety and increase survival over the short term. However, the potential to sustain the species in captivity will depend on the number and sexes of any additional individuals found. Notably, the Española Island tortoise species (*C. hoodensis*) was able to recover to over 3000 individuals from just 12 surviving females and 3 males through a successful long-term captive breeding program^32–34^. Could this same success be achieved with fewer founders? Fernanda has much higher heterozygosity than any of the Española tortoises and if any additional Fernandina individuals have similarly high levels, it could help compensate for a smaller number of potential founders. However, a situation could arise similar to that of the Pinta Island tortoise dubbed “Lonesome George” who failed to breed in captivity and thus was the last of his species^35^.

Examples of other rediscovered species once thought to be extinct highlight the precarious situation of the Fernandina tortoise. Black footed ferrets have successfully rebounded from seven founding individuals, but this was achieved only with substantial human intervention, including the development of advanced techniques for assisted reproduction^36,37^. Similarly, the Bermuda petrel has recovered from 18 breeding pairs through habitat enhancement, the elimination of invasive species, and the reduction of nest competition with other birds^38^. Whether there is enough safe habitat on Fernandina Island to support a larger population of repatriated tortoises is uncertain and will be an important consideration in their future management.

Whether Fernanda is the “endling”^39^ of her species or not, she represents an exciting discovery that engenders hope that even long unseen species may yet survive. The future of the Fernandina tortoise species depends on the outcomes of further searches of this remote and difficult-to-explore island that could result in the discovery of yet more Fernandina tortoises.

## Methods

### Data collection from the Fernandina tortoises

We extracted DNA from a femur of the sole *C. phantasticus* specimen (CAS catalog #8101) which was obtained from the California Academy of Sciences collection. In a dedicated ancient DNA facility at Yale University, a Dremel rotary tool with a cutting blade was used to excise a small wedge of bone (~200 mg), targeting the mid-point in the femur where the bone is densest. The wedge of bone was pulverized in a tube submerged in liquid nitrogen using a Spex 6770 freezer mill. The bone was then demineralized by incubating overnight at 56 °C in a solution of 0.5 M EDTA pH 8.0, 10% SDS, and Proteinase K. DNA was recovered from the resulting lysate using a Qiagen MinElute column. The lysate was mixed with 5× volumes of PB buffer (Qiagen) and centrifuged through the MinElute column. The column was washed twice with PE buffer, before the DNA was eluted using 50 μl of ultra-pure water, warmed to 56 °C.

A small sample of blood (~1 mL) was collected from the brachial artery of a front limb of the living Fernandina tortoise (“Fernanda”). The research was approved by Yale’s Institutional Animal Care and Use Committee (2020–20346). The blood sample was collected under the permit MAE-DNB-CM-2016–0060-M-0003 from the Ecuador Ministry of the Environment, and imported under CITES permit 20US209142/9. The blood was mixed with 3 mL of Longmire Lysis buffer^40^ and stored at 4 °C. DNA was extracted from the blood using a DNeasy Blood and Tissue Kit (Qiagen) according to the manufacturer’s protocol.

For both tortoises, the DNA was prepared into Illumina sequencing libraries by the Yale Center for Genome Analysis, and then sequenced on an Illumina NovaSeq S4.

### Sequence processing and alignment

Sequences were trimmed and aligned to the *C. abingdonii* reference nuclear and mitochondrial genomes assembly ASM359739v1^22^, using the BAM pipeline in PALEOMIX version 1.2.14^41^. In a previous study, analyses were performed to assess whether the use of an in-group reference genome impacted alignment quality, and it was found to not be an issue among Galapagos giant tortoises^12^. PALEOMIX is a wrapper program that employs other bioinformatic tools, including ADAPTERREMOVAL version 2.3.1^42^, to trim, BWA mem version 0.7.17^43^, to align, PICARD *MarkDuplicates* (version 2.6.0, http://broadinstitute.github.io/picard/) and *paleomix rmdup_collapsed* to remove PCR duplicates, and GATK IndelRealigner^44^ to realign around indels. For CAS 8101 an additional step was included in the pipeline to rescale the quality scores of bases that were potentially the result of postmortem DNA damage using MAPDAMAGE 2.0^45^. The BAM files were filtered for a minimum mapquality score of 30, retaining only primary alignments and with an insert size between paired end reads of less than 800 bp using BAMTOOLS version 2.5.1^46^. Regions of the nuclear genome that are potentially repetitive were filtered out using mask files based on mapability (generated using the program SEQBUILITY https://github.com/lh3/misc), and known repetitive elements identified by REPEATMASKER^47^. Contigs in the nuclear genome that are less than 100 kb in length were removed, as they tended to have lower mean mapping quality scores. In total, 2598 nuclear genome contigs were retained, with a total length of 2,226,678,034 bp that is equal to 96.8% of the total genome length.

### Nuclear genome analyses

We analyzed the nuclear genome BAM files for the two Fernandina individuals along with data from 37 other Galapagos giant tortoise genomes, which included three representatives from all 11 of the living species, including three individuals each from the two lineages with *C. becki* (PBL and PBR) (data from Jensen, et al.^12^, NCBI Bioproject PRJNA761229), plus one representative from the extinct Pinta Island species (*C. abingdonii*, data from Quesada, et al.^22^). The *C. abingdonii* individual (the tortoise known as “Lonesome George”) is the one from which the reference genome was constructed^22^, so in order to generate a dataset with equivalent coverage to the other individuals we used only a subset of the Illumina short reads available (NCBI SRA accessions SRR6950587, SRR6950589, and SRR6950615). For phylogenetic analyses, an outgroup, the Chaco tortoise (*C. chilensis*, data from Jensen, et al.^12^, NCBI SRA BioSample SAMN24582572) was also aligned to the *C. abingdonii* genome and used. The geographic locations of each lineage are indicated in Fig. 1, sequencing depths are presented in Supplementary Data 1.

The BAM files for all individuals were used as input to detect variants and call genotypes using BCFTOOLS mpileup/call^48^ which were filtered using VCFTOOLS^49^. Only genotype calls supported by a minimum depth of six reads with a genotype quality score of >17 were retained, and only loci with no missing data with a maximum mean coverage within one standard deviation of the mean coverage across loci were retained. This dataset of 716,435,660 bp, including invariant sites, was used to calculate observed heterozygosity using VCFTOOLS. Invariant sites were then filtered out, to retain only sites with a minor allele count of 1, and the–indep-pairwise function in PLINK v1.9^50^ was used to prune out loci in linkage disequilibrium (LD) using a sliding window size of 50 kb, step size of 5 loci, and *r*^2^ threshold of 0.5. In addition, we calculated heterozygosity using the single-sample SFS approach implemented in ANGSD, including downsampling our read depth and using only transversions (see Supplemental text for detailed methods).

To assess the ancestry and genetic affinities of the two Fernandina individuals, we used PCA, implemented in PLINK v2.0 using the–pca var-wts option, and plotted in R.

### Nuclear phylogenetic analyses

Whole genome sequence fasta files were created from the mapped-read BAM files using ANGSD^51^. Through this method, the base at each site is chosen with the highest number of reads, and indels are skipped. To test for the possible effects of sequence length and linkage in the phylogenetic analyses we created different subsets of data and performed phylogenetic analyses on each of them.

For each set, we randomly selected a starting point on each contig of the assembly between site 100,000 and 1,000,000 to avoid starting too close to a potential telomere. From there, we extracted a fixed length of sequence (10 or 100 kb), skipped a length of sequence (100 kb or 1 Mb) of sequence, extracted the following fixed length of sequence, and continued this pattern through the end of the contig. The same coordinates were then used to extract homologous sequences from all other samples. Because sequences were extracted using coordinates of the reference genome, re-alignment of the extracted sequences was not necessary. This process was repeated to create separate aligned sequence datasets of 10 kb or 100 kb in length, and separated by 100 kb or 1 Mb to test for the effects of sequence length and possible linkage in the phylogenetic analyses. The python script for generating these sequences is available on our GitHub repository (https://github.com/sjgaughran/tortoise-phylogenomics).

Each alignment was filtered using the AMAS package^52^ to retain alignments with more than 5 parsimony informative sites, less than 10% missing data, and a GC-content greater than 30% but less than 70%. Maximum likelihood trees were then constructed for each alignment using RAxML^23^ with the GTR-GAMMA model, 20 bootstraps (-N 20), and assigning the *C. chilensis* tortoise sequence as the outgroup. The resulting gene trees were concatenated and used as input for ASTRAL^24^ to find the best species tree from the gene trees, with branch support reflecting quartet support (i.e., the amount of gene tree conflict around the branch).

To calculate genetic distance, we generated custom python scripts that calculated the number of pairwise differences between individuals in each alignment, divided by the number of sites for which there was no missing data (‘N’) for that pair of individuals. We then averaged this across all alignments for each data set. Because genetic distances between individuals are higher in populations with higher genetic diversity, we used a measure of pairwise genetic distance that accounts for the average heterozygosity of the individuals being compared. To account for differences in heterozygosity across individuals, for each pairwise comparison we took the average of the heterozygosity between the two individuals and divided the absolute pairwise genetic distance by this average heterozygosity. This normalized genetic distance can be described as:

Equation 1.

1 $${{{{{\rm{Distance}}}}}}=\,\frac{\frac{1}{n}\mathop{\sum }\limits_{i=1}^{n}\frac{{a}_{i}}{{S}_{i}}}{\frac{{h}_{1}+{h}_{2}}{2}}$$

where *n* is the number of genomic segments, *a*_*i*_ is the number of pairwise differences between the two individuals for segment *i*, *S*_*i*_ is the number of non-N base pairs in segment *i*, *h*_1_ is the per-base pair genome-wide heterozygosity of one individual and *h*_2_ is the per-base pair genome wide heterozygosity of the other individual. The scripts for calculating this distance and plotting the comparisons are available on our GitHub repository (https://github.com/sjgaughran/tortoise-phylogenomics).

### Mitochondrial genome analyses

We analyzed the mitochondrial genome BAM files for the two Fernandina individuals along with the same representatives of the 12 living lineages and *C. abingdonii* as for the nuclear analyses, with the addition of one other *C. abingdonii* individual (historical specimen CAS 8112, collected in 1906 from the wild) and one individual of *C. niger* (historical specimen 46606 from the Harvard Museum of Comparative Zoology, “MCZ”) from Floreana Island. Mitochondrial genomes for these two additional historical individuals were obtained using low-coverage whole genome sequencing, following the same DNA extraction, library preparation, sequencing, and analysis methods as described for CAS 8101. For CAS 8112 and MCZ 46606 coverage of the nuclear genome was too low to include them in those analyses, but coverage of the mitochondrial genome was 138× and 13×, respectively (Supplemental Table 2). The mitochondrial BAM files were input into BCFTOOLS mpileup/call with the -c option to write the consensus allele. The VCF file was converted to Phylip format and aligned with mitochondrial genome sequences for the outgroup taxon *C. chilensis* (LT599484).

The mitochondrial genomes were realigned with MUSCLE^53^, and concatenated into a super matrix for the determination of appropriate substitution models. The final super matrix of 15,522 bases included 13 protein coding genes, 22 tRNAs, and 2 ribosomal RNAs (Table S2).

PartitionFinder2 (PF) v.2.1^54^ was used to find the best-fit partitioning scheme and the best nucleotide substitution model for each partition (Table S3). The alignment of mitogenomes was subdivided into 63 predefined blocks; 39 of them correspond to each codon position for the 13 coding genes, 2 to the ribosomal genes (12S rRNA and 16S rRNA), and 22 to tRNAs. The analysis was run twice in order to find the best-fit partitioning scheme and evolutionary models for each downstream analysis according to the models that can be implemented in software that we used in phylogenetic analyses (RAxML, MrBayes^55^). In all analyses, linked branch lengths and greedy algorithm were selected to search for the best-fit solutions, and the model selection was based on the Bayesian Information Criterion (BIC) that is substantially more accurate in finding the true model than AIC/AICc^56^, ignoring the models that contain both gamma distribution and invariable sites^57^.

Bayesian inference (BI) analysis was performed in MrBayes v.3.2.7^55^, performing four runs and using eight sampling chains for each run based on the partition and models revealed in PF2. Each chain ran for 10,000,000 generations, sampling every 5000 generations. To check for convergence and stationarity, we used the plot of the generation versus the log probability of the data (the log likelihood values), the average standard deviation of split frequencies, the average Potential Scale Reduction Factor (PSRF), and the minimum value of Estimated Sample Sizes (ESS). The first 25% of trees were discarded as burn-in. A 50% majority rule consensus tree was then produced from the posterior distribution of trees and the posterior probabilities were calculated as the percentage of samples recovering any particular clade. Posterior probabilities ≥ 0.95 indicate statistically significant support^58^.

Maximum likelihood (ML) analysis was performed using RAxML v.8.2.12. The tree with the best likelihood for each dataset was selected among the 50 ML trees generated on distinct starting trees. Statistical confidence was assessed based on 1000 thorough bootstraps.

### Statistics and reproducibility

Statistical support for gene trees and the mitochondrial trees was assessed as bootstrap values in RAxML for the ML analyses, and as posterior probabilities in MrBayes in the BI analysis. The method for calculating normalized genetic distance as described above was carried out in a custom python script, which is available on our GitHub repository (see “Code Availability”).

### Reporting summary

Further information on research design is available in the Nature Research Reporting Summary linked to this article.

## Supplementary information

Supplemental Material

Supplementary Data 1

Reporting Summary

## Data Availability

Fastq files of the two Fernandina individuals are available on the NCBI SRA as accession numbers SAMN24674816 and SAMN24674817. The mitochondrial genome haplotypes are available on GenBank as accession numbers OM719670-OM719710.

## Code availability

The scripts used in this study are available on our GitHub repository https://github.com/sjgaughran/tortoise-phylogenomics.
