# Supplementary material for: The Galapagos giant tortoise Chelonoidis phantasticus is not extinct
Source: Commun Biol. 2022 Jun 9;5:546. doi: 10.1038/s42003-022-03483-w (PMC9184544; doi:10.1038/s42003-022-03483-w)
Supplement: Supplementary file 1 — Supplemental Material [file 42003_2022_3483_MOESM1_ESM.pdf]

Supplementary Information for:

The Galapagos giant tortoise *Chelonoidis phantasticus* is not extinct

Authors: Evelyn L. Jensen, Stephen J. Gaughran, Nicole A. Fusco, Nikos Poulakakis, Washington Tapia, Christian Sevilla, Jeffreys Málaga, Carol Mariani, James P. Gibbs and Adalgisa Caccone

Supplementary Text

Abstract in Spanish

El estado de la población de tortuga gigante de la isla Fernandina (*Chelonoidis phantasticus*) en el archipiélago de Galápagos ha sido un misterio, con la especie conocida a partir de un solo espécimen colectado en 1906. El descubrimiento en 2019 de una tortuga hembra viviendo en la isla brindó la oportunidad de determinar si la especie aún sigue viva. Mediante la secuenciación de los genomas de ambos individuos y comparándolos con todas las especies de tortugas gigantes de Galápagos que aún viven, aquí demostramos que las dos tortugas conocidas de Fernandina son del mismo linaje pero distintas de todas las demás. La filogenia del genoma completo agrupa a los individuos de Fernandina dentro de un grupo monofilético que contiene todas las especies con morfología de caparazón tipo montura y una especie semimontura. Esta agrupación de las especies montura es contraria a la filogenia del ADN mitocondrial, que ubica a las especies montura en varios clados. Estos resultados implican la continua existencia de un linaje considerado extinto durante mucho tiempo, con un tamaño de población actual conocido de un solo individuo.

Sequencing results

After filtering the BAM files 318,928,748 reads for Fernanda and 204,428,807 reads for Fern 1906 were aligned, resulting in an average coverage of 34x and 22x, respectively. The bone for

the Fern 1906 specimen is exceptionally well preserved, with over 99% of reads originating from endogenous DNA. However, the DNA itself was still highly fragmented however, with an average length of 119 bp, as visualized on a bioanalyzer. When the sequences obtained were trimmed and overlapping forward and reverse reads were collapsed (92% of reads collapsed), there was a mean read length of 176 bp. So, although we observe that there is a high proportion of endogenous DNA, that DNA is still degraded, as expected with the age of the specimen. Additionally, the MapDamage analysis indicated very low rates of DNA damage, with a minimal increase in base misincorporation of C->T overall, and no trend in the misincorporation rate relative to position along a read.

After filtering the SNP variants for minor allele count of 1, allowing no missing data, having a maximum mean depth cut off 1 SD above the mean (here equal to 31.3x) and pruning for LD, a total of 751,800 SNP loci were retained for use in the PCA analysis.

Nuclear genome phylogenetic trees

Because consensus species tree construction can be affected by the number of gene trees, the length of sequence used to create those trees, and non-independence (i.e., linkage) between trees, we created four datasets of genomic segments of different lengths (10kb or 100kb) and separated by different distances (100kb or 1Mb). The number of gene trees in a data set ranged from 306 (100kb segments separated by 1Mb) to 7212 (10kb segments separated by 100kb). We recovered nearly identical topologies across datasets (Figs. 2B, S1–3), although node support was lower in consensus trees created from datasets with fewer gene trees (e.g., Fig. S2–3).

The non-monophyly and poor node support for the Fernandina tortoises when analyzed with the 13 other species was a surprise given the close genetic relationship between these two tortoises and their shared island of origin. Given the low support for other nodes across the tree, we suspected that the recent radiation of these species had led to low sequence divergence and high rates of incomplete lineage sorting (ILS) among species, leading to a high incongruence among gene trees. However, given that both the consensus tree and the carapace morphology of Galapagos giant tortoises support the monophyly of saddleback tortoises, we created four new datasets, using the same filtering parameters described above, but this time only including sequences from Fernanda, the species with saddleback morphology (i.e., the species from San Cristóbal, Pinzón, Española, Pinta, and Fernandina), and the *C. chilensis* outgroup. The consensus trees produced from these four saddleback datasets showed highly congruent topologies (Fig. 2C, Figs. S4–6). Notably, all described species were highly supported monophyletic groups, including the two tortoises from Fernandina. Again, we observe that consensus trees made from fewer and shorter segments have lower node support (Fig. S4) compared to those made from more segments (Fig. S5) or longer segments (Fig. 2C).

None of the nuclear trees place the individuals of *C. becki* into a monophyletic clade. This species, found on Volcano Wolf at the northern end of Isabela Island, is known to consist of two lineages (referred to as PBL and PBR) originating from different colonization events [1], yet our nuclear phylogenies find the PBL lineage itself to be split across major clades in the tree. This placement may have been influenced by recent gene flow into *C. becki* as a result of humans transporting tortoises or represent historical admixture.

Mitogenome phylogenetic trees

The best-fit partitioning scheme for each downstream analysis, and the selected nucleotide substitution models are given in Table S3. The ML and BI analyses resulted in phylogenetic trees with lnL= -27,213.56 and lnL= -26,919.10 (harmonic mean), respectively. All MCMC diagnostic metrics indicated that the iterations of BI analysis reached convergence and stationarity. The average standard deviation of split frequencies was smaller than 0.01, the plot of generation versus log-likelihood of the data had characteristic "white-noise" morphology after burn-in. The PSRF values were near 1.00 (range 0.999 – 1.000) and the minimum ESS values were well over 100 (the minimum value was 1,072.40).

Tree topologies from the BI and ML analyses were identical (Fig. S7), having high posterior probabilities (ps) and bootstrap support (bs) values for all nodes (pp ≥ 0.99, bs ≥ 82), except for the sister group relationship of *C. becki* and *C. darwini* with *C. abingdonii*, *C. hoodensis*, *C. chathamensis*, and *C. donfaustoi* (pp = 0.89 and bs = 69). Fernanda is clustered with the extinct species from Floreana island (*C. niger*), whereas Fern 1906 is clustered with the *C. porteri* lineage from East Santa Cruz.

There are important points of discordance between the nuclear phylogenies and that based on the mitochondrial genome. Most noticeably, the saddleback species form a monophyletic clade in the nuclear trees, which is in discordance with trees based on the mitochondrial genome, where they are dispersed across the tree (see Supplementary Text, Fig. S7, [2]). Additionally, the two species from Santa Cruz Island are sister taxa on the nuclear trees, whereas they are in different clades in the mitochondrial tree. These findings have important implications for our

understanding of how the saddleback morphology evolved in Galapagos tortoises, and the phylogeographic history of the radiation.

Explorations of heterozygosity estimates

The average depth of sequencing coverage varied from 9.5X to 34X across our samples (Supplemental File 2). Because estimates of heterozygosity can be affected by coverage, we used ANGSD [3] to down-sample each BAM file in our sample set to an average of 9.5X coverage with option -downSample C, where C is 9.5 divided by the sample coverage. We then re-calculated genome-wide heterozygosity in ANGSD, using the 1 sample SFS Estimation method.

We found that in our sample set, there was no correlation between depth of coverage and heterozygosity estimate using either VCFtools or ANGSD (Supplemental File 2). Furthermore, although the estimates from ANGSD and VCFtools were different, the estimates were highly correlated. Finally, we found that down-sampling the BAMS to 9.5X led to heterozygosity being underestimated by an average of 4.6%, which is small compared to the difference seen between down-sampled individuals. For example, the difference in heterozygosity between Fernanda and other tortoises ranges from 7-200%. These results suggest that qualitative comparisons of heterozygosity are reliable in our sample set, even when depth of coverage differs among samples.

In addition, transitions can occur at artificially higher rates in ancient samples due to cytosine deamination in ancient DNA samples. Because sample CAS8101 is a museum specimen, we re-estimated heterozygosity by removing transitions in ANGSD (-noTrans 1). The estimate of heterozygosity for CAS8101 was marginally reduced relative to modern samples (Supplemental

File 2). However, it was still the sample with the second highest genome-wide heterozygosity, even by this measure.

Supplementary References

1 Garrick, R. C. *et al.* Lineage fusion in Galapagos giant tortoises. *Mol Ecol* **23**, 5276-5290, doi:10.1111/mec.12919 (2014).

2 Poulakakis, N. *et al.* Colonization history of Galapagos giant tortoises: Insights from mitogenomes support the progression rule. *Journal of Zoological Systematics and Evolutionary Research* **58**, 1262-1275, doi:10.1111/jzs.12387 (2020).

3 Korneliussen, T. S., Albrechtsen, A. & Nielsen, R. ANGSD: Analysis of Next Generation Sequencing Data. *BMC Bioinformatics* **15**, 356, doi:10.1186/s12859-014-0356-4 (2014).

Supplementary Figures

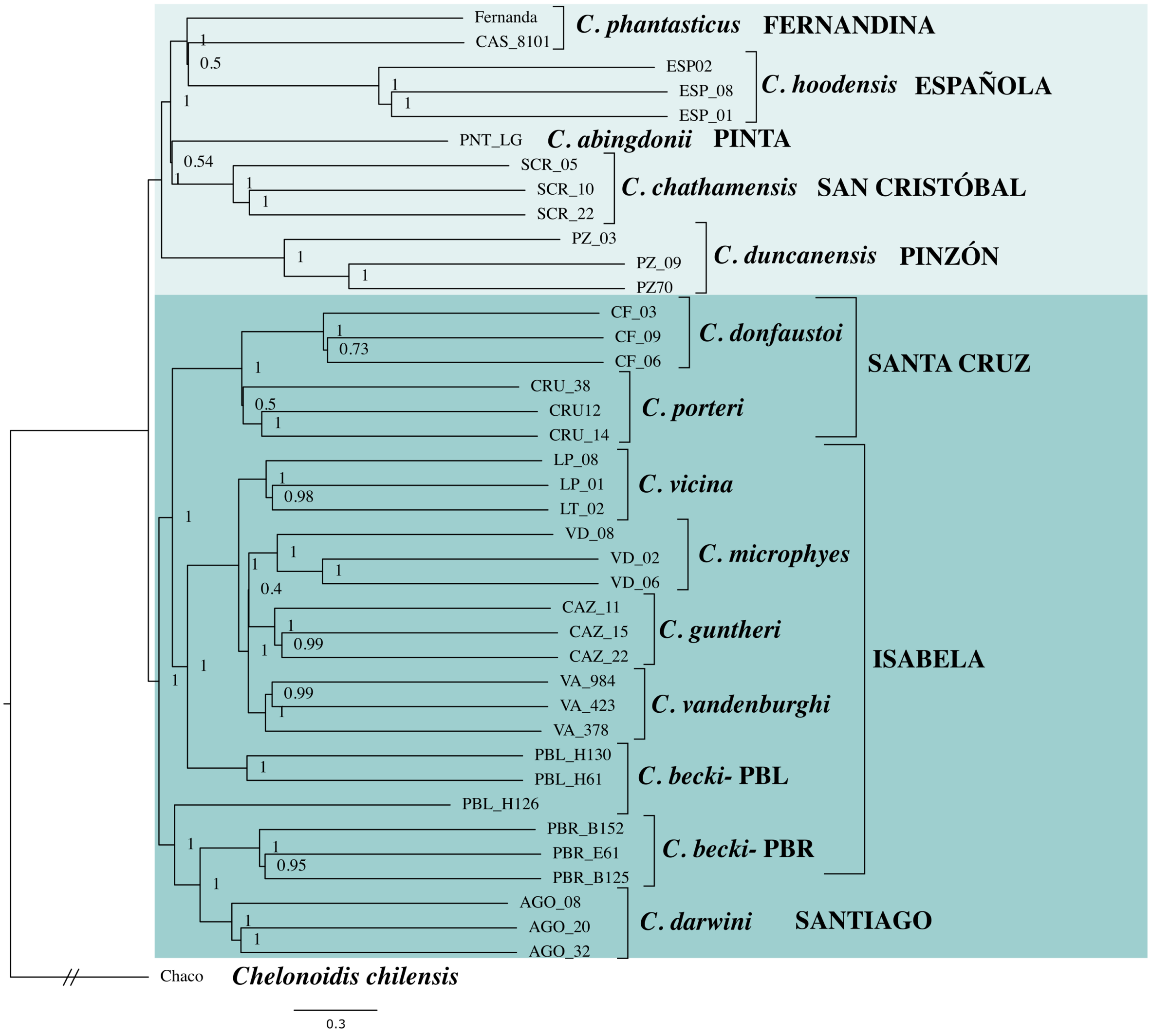

Fig. S1.

Astral consensus tree for 14 species of Galapagos giant tortoise (*Chelonoidis sp.*) created from 7121 maximum likelihood trees built from 10 kb segments of the genome, each spaced 100 kb apart. Species names are in italics, island names are in capital letters. The lighter box highlights the clade with predominantly saddleback carapace morphology, the darker box indicates the clade with predominantly domed morphology. Values on the nodes indicate posterior probabilities.

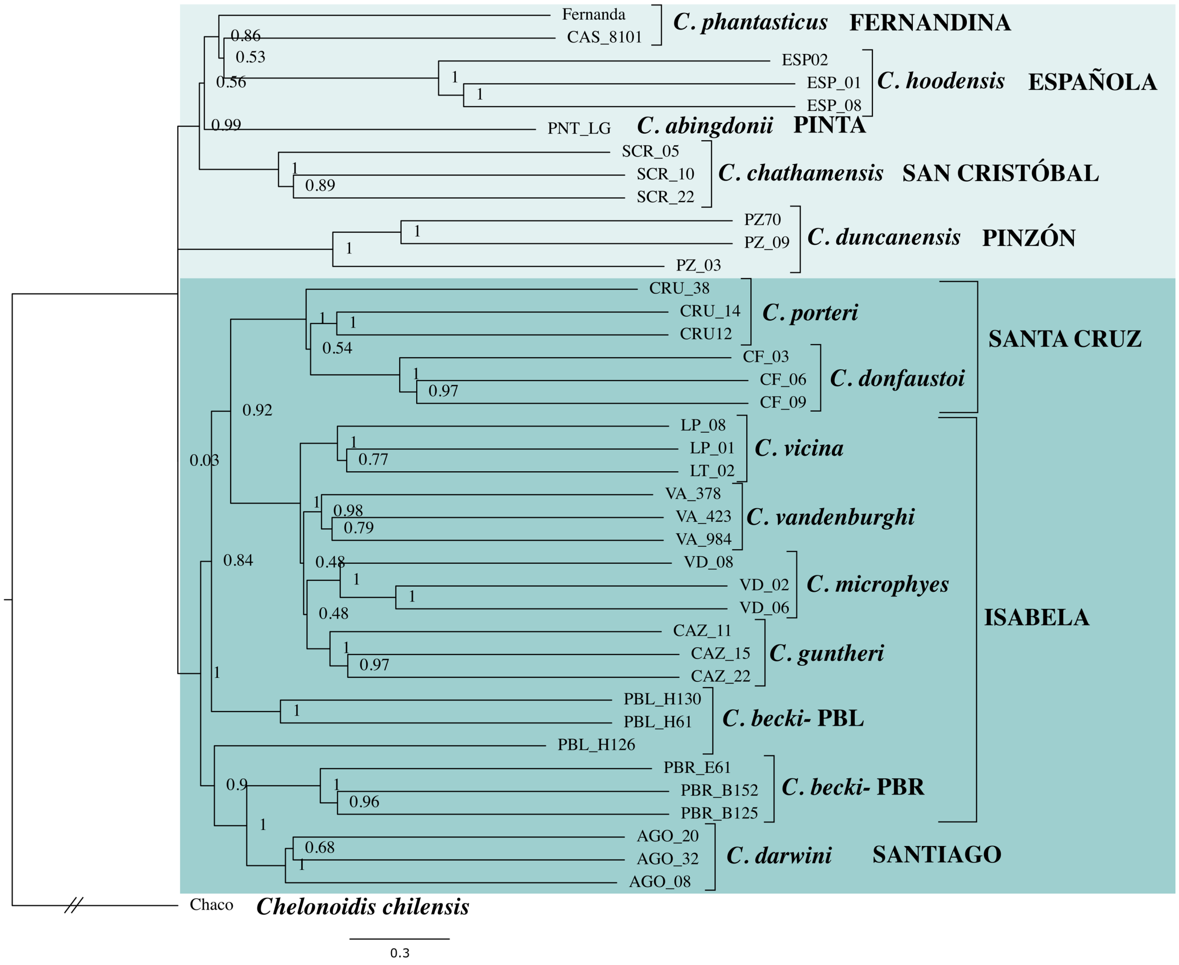

Fig. S2.

Astral consensus tree for 14 species of Galapagos giant tortoise (*Chelonoidis sp.*) created from 1130 maximum likelihood trees built from 10 kb segments of the genome, each spaced 1Mb apart. Species names are in italics, island names are in capital letters. The lighter box highlights the clade with predominantly saddleback carapace morphology, the darker box indicates the clade with predominantly domed morphology. Values on the nodes indicate posterior probabilities.

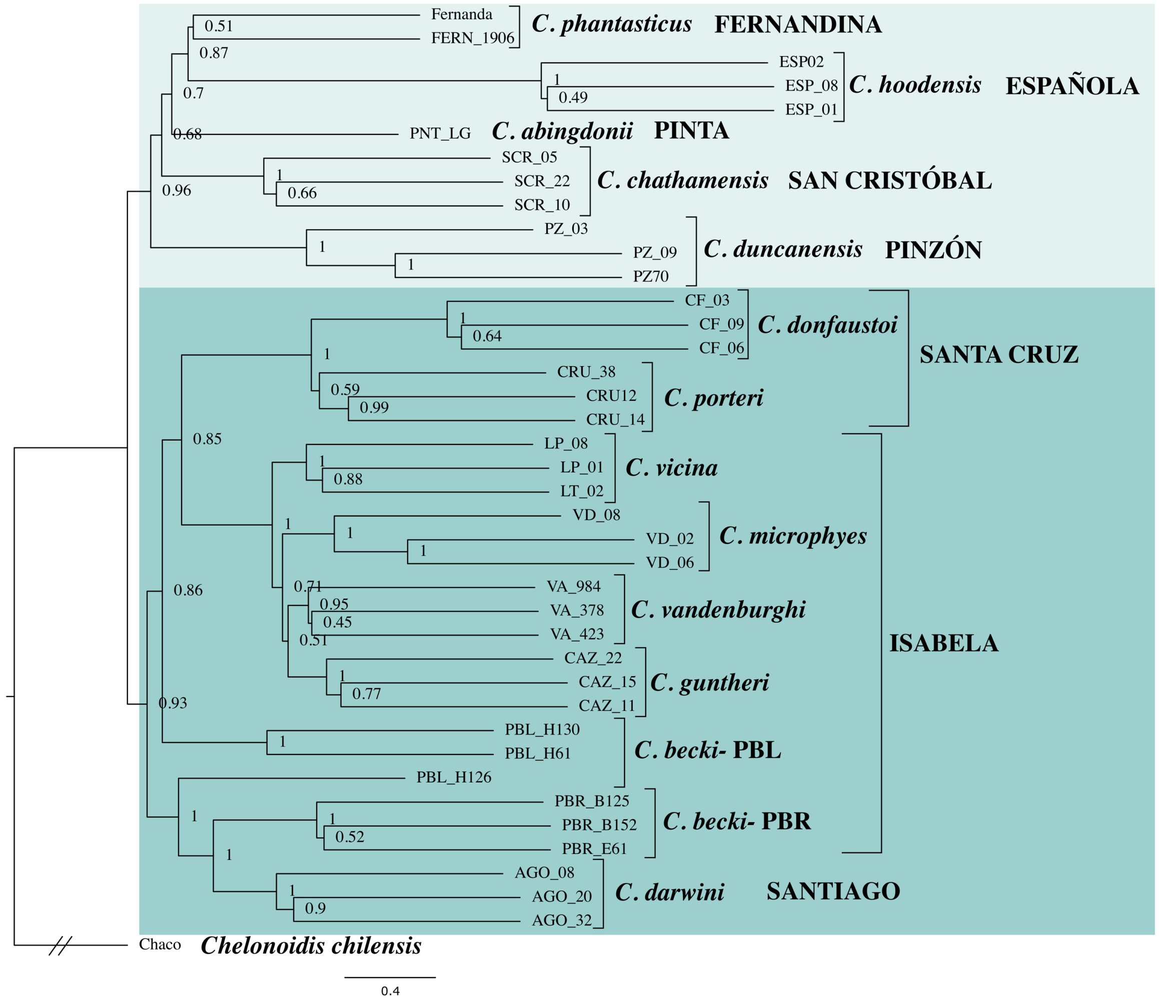

Fig. S3.

Astral consensus tree for 14 species of Galapagos giant tortoise (*Chelonoidis sp.*) created from 306 maximum likelihood trees built from 100 kb segments of the genome, each spaced 1Mb apart. Species names are in italics, island names are in capital letters. The lighter box highlights the clade with predominantly saddleback carapace morphology, the darker box indicates the clade with predominantly domed morphology. Values on the nodes indicate posterior probabilities.

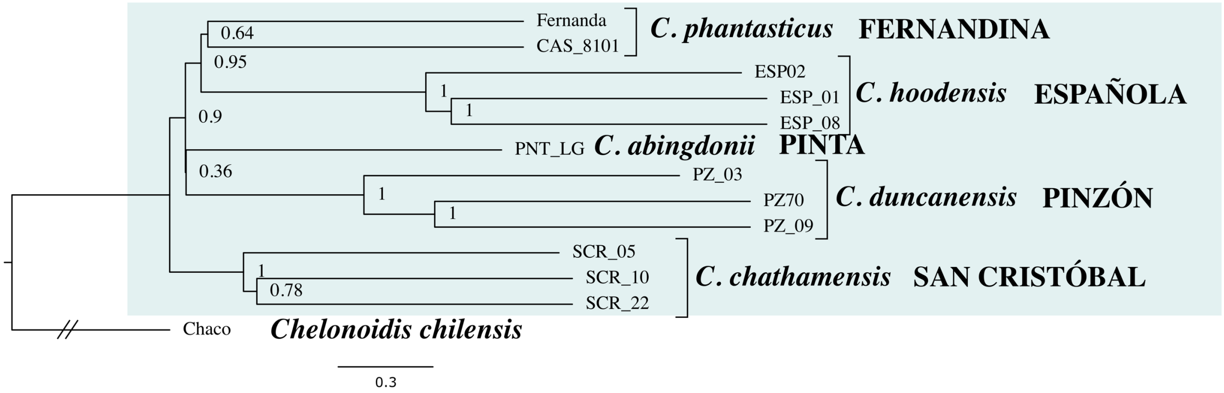

Fig. S4

Astral consensus tree of the predominantly saddleback species of Galapagos giant tortoise, created from 1016 maximum likelihood trees built from 10kb segments of the genome, each spaced 1Mb apart. Species names are in italics, island names are in capital letters. Values on the nodes indicate posterior probabilities.

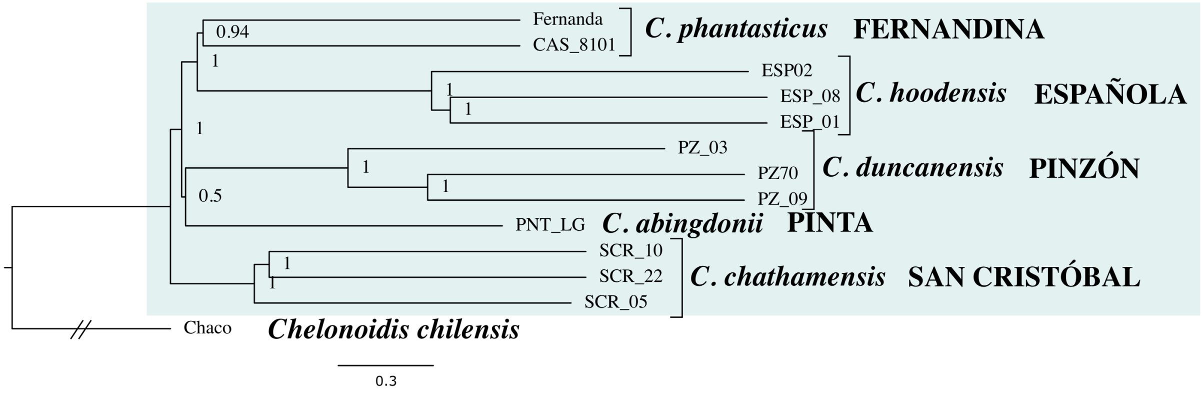

Fig. S5

Astral consensus tree of the predominantly saddleback species of Galapagos giant tortoise, created from 7090 maximum likelihood trees built from 10kb segments of the genome, each spaced 100kb apart. Species names are in italics, island names are in capital letters. Values on the nodes indicate posterior probabilities.

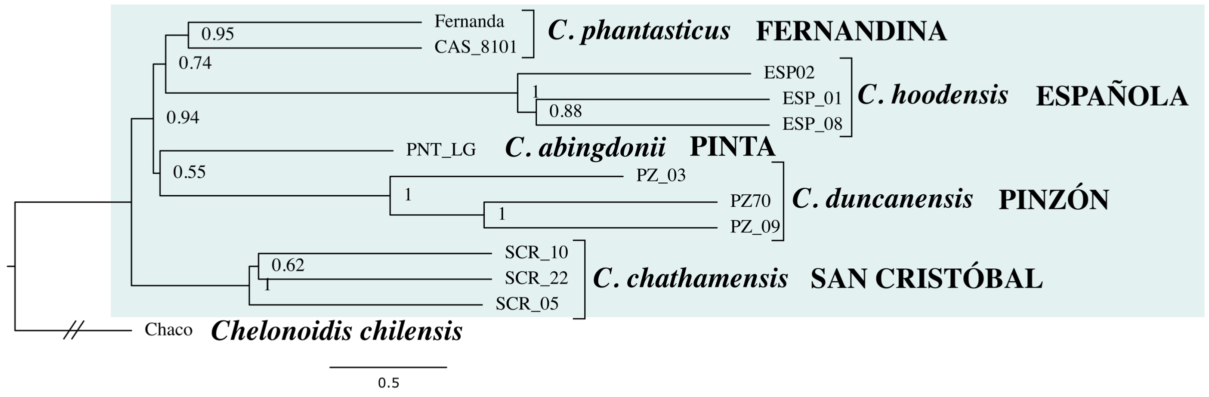

Fig. S6

Astral consensus tree of the predominantly saddleback species of Galapagos giant tortoise, created from 264 maximum likelihood trees built from 100kb segments of the genome, each spaced 1Mb apart. Species names are in italics, island names are in capital letters. Values on the nodes indicate posterior probabilities.

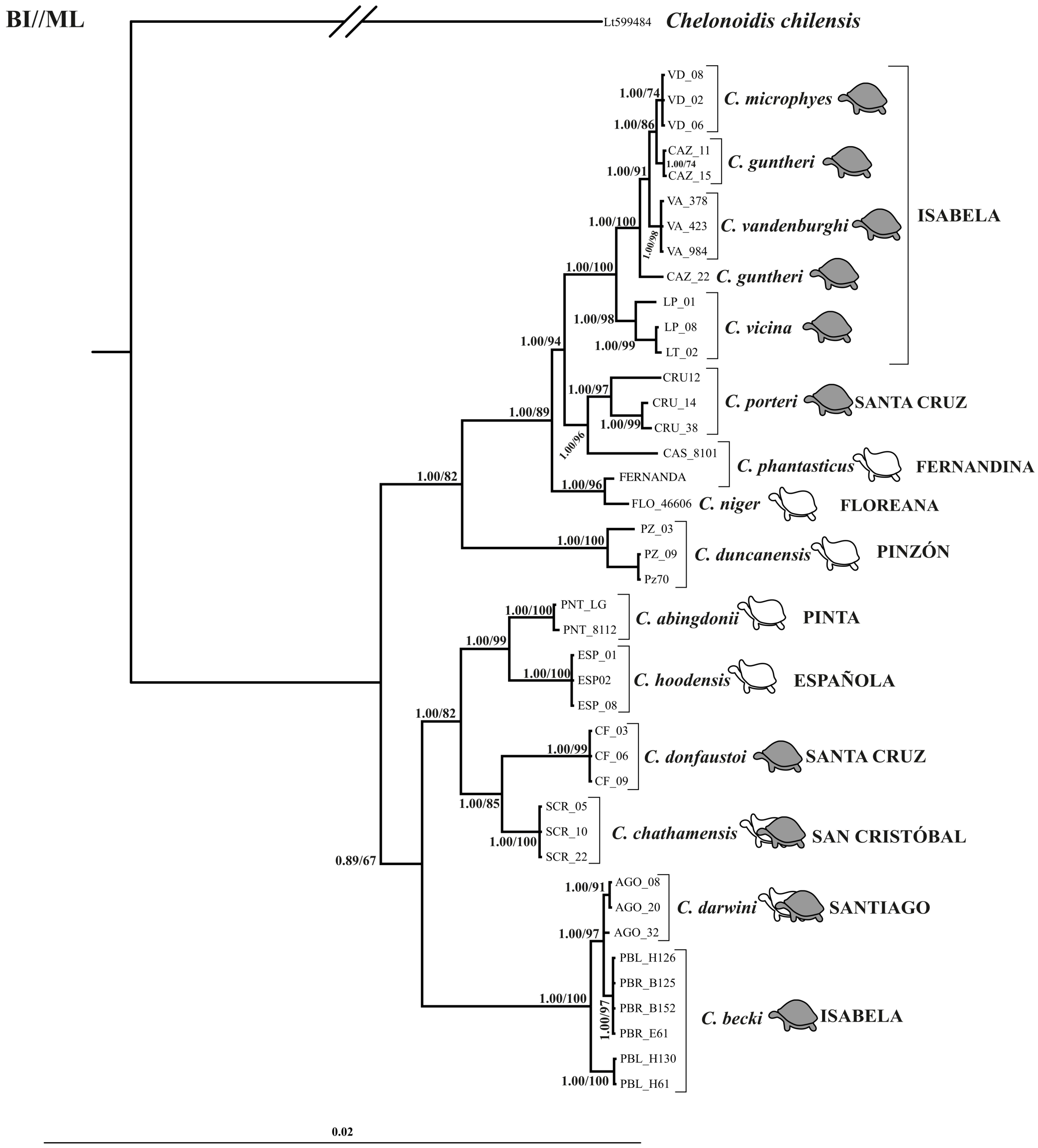

Fig. S7.

Bayesian Inference tree based on the complete mitochondrial genomes of Galapagos giant tortoise and outgroup taxa. The posterior probabilities for BI and bootstrap support for ML are

given on branches (ML/BI). Tortoise icons indicate the morphology of the species, either domed (grey), saddleback (white) or semi-saddleback (indicated with both icons present).

Supplementary Tables

Table S1.

Mean autosomal heterozygosity estimated using VCFtools within each species of Galapagos giant tortoise and the two individuals from Fernandina Island.

Island	Species		Heterozygosity
Fernandina	*C. phantasticus*	Fernanda	0.00052
		CAS 8101	0.00053
Española	*C. hoodensis*		0.00027
Isabela	*C. becki -PBL*		0.00046
	C. becki -PBR		0.00036
	C. guntheri		0.00033
	C. microphyes		0.00033
	C. vandenburghi		0.00034
	C. vicina		0.00031
Pinta	*C. abingdonii*		0.00015
Pinzón	*C. duncanensis*		0.00036
San Cristóbal	*C. chathamensis*		0.00043
Santa Cruz	*C. porteri*		0.00043
	C. donfaustoi		0.00032
Santiago	*C. darwini*		0.00044

Table S2.

Partitioning of the mitochondrial genomes used in this study based on the gene fragment and codon position for the coding genes.

Fragment	Partition	Length (bp)
tRNA-Phe	trnF = 1-70	70
ssrRNA	rrnS = 71-1044	974
tRNA-Val	trnV = 1045-1114	70
lsrRNA	rrnL = 1115-2722	1608
tRNA-Leu2	trnL2 = 2723-2799	77
ND1	ND1_codon1 = 2800-3770\3	971
	ND1_codon2 = 2801-3770\3	
	ND1_codon3 = 2802-3770\3	
tRNA-Ile	trnI = 3771-3839	69
tRNA-Gln	trnQ = 3840-3909	70
tRNA-Met	trnM = 3910-3978	69
ND2	ND2_codon1 = 3979-5017\3	1039
	ND2_codon2 = 3980-5017\3	
	ND2_codon3 = 3981-5017\3	
tRNA-Trp	trnW = 5018-5094	77
tRNA-Ala	trnA = 5095-5165	71
tRNA-Asn	trnN = 5166-5265	100
tRNA-Cys	trnC = 5266-5331	66
tRNA-Tyr	trnY = 5332-5403	72
COX1	cox1_codon1 = 5404-6942\3	1539
	cox1_codon2 = 5405-6942\3	
	cox1_codon3 = 5406-6942\3	
tRNA-Ser2	trnS2 = 6943-7013	71

tRNA-Asp	trnD = 7014-7083	70
COX2	cox2_codon1 = 7084-7775\3	692
	cox2_codon2 = 7085-7775\3	
	cox2_codon3 = 7086-7775\3	
tRNA-Lys	trnK = 7776-7846	71
ATP8	atp8_codon1 = 7847-8001\3	155
	atp8_codon2 = 7848-8001\3	
	atp8_codon3 = 7849-8001\3	
ATP6	atp6_codon1 = 8002-8684\3	683
	atp6_codon2 = 8003-8684\3	
	atp6_codon3 = 8004-8684\3	
COX3	cox3_codon1 = 8685-9468\3	784
	cox3_codon2 = 8686-9468\3	
	cox3_codon3 = 8687-9468\3	
tRNA-Gly	trnG = 9469-9536	67
ND3	ND3_codon1 = 9537-9886\3	350
	ND3_codon2 = 9538-9886\3	
	ND3_codon3 = 9539-9886\3	
tRNA-Arg	trnR = 9887-9956	70
ND4L	ND4l_codon1 = 9957-10246\3	290
	ND4l_codon2 = 9958-10246\3	
	ND4l_codon3 = 9959-10246\3	
ND4	ND4_codon1 = 10247-11625\3	1379
	ND4_codon2 = 10248-11625\3	
	ND4_codon3 = 10249-11625\3	
tRNA-His	trnH = 11626-11695	70
tRNA-Ser	trnS = 11696-11770	75
tRNA-Leu	trnL = 11771-11842	72
ND5	ND50_codon1 = 11843-13645\3	1803
	ND50_codon2 = 11844-13645\3	

	ND50_codon3 = 11845-13645\3	
ND6	ND6_codon1 = 13646-14167\3	522
	ND6_codon2 = 13647-14167\3	
	ND6_codon3 = 13648-14167\3	
tRNA-Glu	trnE = 14168-14239	72
cytB	cytb_codon1 = 14240-15383\3	1144
	cytb_codon2 = 14241-15383\3	
	cytb_codon3 = 14242-15383\3	
tRNA-Thr	trnT = 15384-15453	70
tRNA-Pro	trnP = 15454-15522	68

Table S3.

Partitioning schemes and best-fit models of sequence evolution selected in PartitionFinder2 (PF) for downstream analyses.

Partition Scheme from PF		Model of evolution	Length of partition	Fragments of partition
MrBayes	1	HKY+I	6048	trnF, trnD, trnW, trnE, trnP, trnT, trnS2, trnL1, trnK, trnC, trnR, ND50_codon1, trnA, trnQ, trnV, cytb_codon1, trnS1, trnY, atp6_codon1, ND4_codon1, rrnS, ND6_codon2, ND2_codon1, rrnL, trnG, trnH
	2	K80+I	1742	cox2_codon1, trnN, cox1_codon1, cox3_codon1, ND4l_codon1, ND1_codon1, trnM, trnL2, trnI
	3	HKY+I	2225	atp8_codon2, ND3_codon2, ND50_codon2, atp6_codon2, ND2_codon2, ND1_codon2, ND4l_codon2, ND4_codon2
	4	HKY+G	2823	ND4_codon3, ND50_codon3, cytb_codon3, atp8_codon3, cox3_codon3, atp6_codon3, ND1_codon3, ND6_codon1, ND2_codon3
	5	HKY+I	1386	cytb_codon2, cox1_codon2, cox3_codon2, cox2_codon2
	6	HKY+G	839	cox2_codon3, cox1_codon3, ND4l_codon3
	7	HKY+G	459	ND6_codon3, ND3_codon3, ND3_codon1, atp8_codon1
RAxML	1	GTR+I+G	6175	ND3_codon1, atp8_codon1, ND6_codon3, ND50_codon1, trnS2, trnS1, ND3_codon3, trnH, trnR, trnG, trnC, trnT, trnM, trnF, trnD, atp8_codon2, trnA, trnE, trnW, trnV, trnQ, ND2_codon1, atp6_codon1, trnP, ND6_codon2, trnL1, trnY, ND4_codon1, rrnL, rrnS
	2	GTR+I+G	2126	trnI, cox1_codon1, ND4l_codon1, ND1_codon1, trnN, cyt_codon1, cox3_codon1, trnK, trnL2, cox2_codon1
	3	GTR+I+G	3559	ND3_codon2, cox3_codon2, cox2_codon2, ND1_codon2, ND4l_codon2, ND2_codon2, cox1_codon2, cyt_codon2, ND50_codon2, ND4_codon2, atp6_codon2
	4	GTR+G	3662	ND50_codon3, ND2_codon3, ND6_codon1, ND1_codon3, cox3_codon3, cyt_codon3, atp8_codon3, ND4l_codon3, ND4_codon3, cox1_codon3, atp6_codon3, cox2_codon3
