# Supplementary material for: The Galapagos giant tortoise Chelonoidis phantasticus is not extinct
Source: Commun Biol. 2022 Jun 9;5:546. doi: 10.1038/s42003-022-03483-w (PMC9184544; doi:10.1038/s42003-022-03483-w)
Supplement: Supplementary file 3 — Reporting Summary [file 42003_2022_3483_MOESM3_ESM.pdf]

nature portfolio

Corresponding author(s): Evelyn Jensen

Last updated by author(s): Feb 22, 2022

Reporting Summary

Nature Portfolio wishes to improve the reproducibility of the work that we publish. This form provides structure for consistency and transparency in reporting. For further information on Nature Portfolio policies, see our Editorial Policies and the Editorial Policy Checklist.

Statistics

For all statistical analyses, confirm that the following items are present in the figure legend, table legend, main text, or Methods section.

n/a	Confirmed	
☐	☒	The exact sample size (n) for each experimental group/condition, given as a discrete number and unit of measurement
☒	☐	A statement on whether measurements were taken from distinct samples or whether the same sample was measured repeatedly
☐	☒	The statistical test(s) used AND whether they are one- or two-sided<br>*Only common tests should be described solely by name; describe more complex techniques in the Methods section.*
☒	☐	A description of all covariates tested
☐	☒	A description of any assumptions or corrections, such as tests of normality and adjustment for multiple comparisons
☐	☒	A full description of the statistical parameters including central tendency (e.g. means) or other basic estimates (e.g. regression coefficient) AND variation (e.g. standard deviation) or associated estimates of uncertainty (e.g. confidence intervals)
☒	☐	For null hypothesis testing, the test statistic (e.g. F, t, r) with confidence intervals, effect sizes, degrees of freedom and P value noted<br>*Give P values as exact values whenever suitable.*
☐	☒	For Bayesian analysis, information on the choice of priors and Markov chain Monte Carlo settings
☒	☐	For hierarchical and complex designs, identification of the appropriate level for tests and full reporting of outcomes
☒	☐	Estimates of effect sizes (e.g. Cohen's d, Pearson's r), indicating how they were calculated

Our web collection on statistics for biologists contains articles on many of the points above.

Software and code

Policy information about availability of computer code

Data collection	No software was used.
Data analysis	Paleomix, version 1.2.14<br>Adapterremoval, version 2.3.1<br>BWA, version 0.7.17<br>Picard, version 2.6.0<br>GATK<br>Mapdamage, version 2.0<br>Bamtools, version 2.5.1<br>Seqbuilty, https://github.com/lh3/misc<br>Repeatmasker<br>BCFtools, version 1.9<br>VCFtools, version 0.1.16<br>Plink, version 1.9<br>Plink, version 2.0<br>ANGSD, version 0.93<br>Custom script: https://github.com/sjgaughran/tortoise-phylogenomics<br>AMAS<br>RAxML<br>Astral<br>Muscle<br>PartitionFinder, Version 2.1<br>MrBayes, version 3.2.7

For manuscripts utilizing custom algorithms or software that are central to the research but not yet described in published literature, software must be made available to editors and reviewers. We strongly encourage code deposition in a community repository (e.g. GitHub). See the Nature Portfolio guidelines for submitting code & software for further information.

Data

Policy information about availability of data

All manuscripts must include a data availability statement. This statement should provide the following information, where applicable:

- Accession codes, unique identifiers, or web links for publicly available datasets
- A description of any restrictions on data availability
- For clinical datasets or third party data, please ensure that the statement adheres to our policy

Fastq files of the two Fernandina individuals are available on the NCBI SRA as accession numbers SAMN24674816 and SAMN24674817. The mitochondrial genome haplotypes are available on GenBank as accession numbers OM719670-OM719710.

Field-specific reporting

Please select the one below that is the best fit for your research. If you are not sure, read the appropriate sections before making your selection.

☐ Life sciences ☐ Behavioural & social sciences ☒ Ecological, evolutionary & environmental sciences

For a reference copy of the document with all sections, see nature.com/documents/nr-reporting-summary-flat.pdf

Ecological, evolutionary & environmental sciences study design

All studies must disclose on these points even when the disclosure is negative.

Study description	Whole genome sequences were collected via shotgun sequencing from one living Galapagos giant tortoise, and one museum speciemen, and compared to an existing dataset of whole genome sequences.
Research sample	1 living tortoise= Fernanda<br>1 museum tortoise= California Academy of Sciences specimen 8101
Sampling strategy	The sample size of two reflects that these are the only known individuals of their species.
Data collection	Blood was collected from Fernanda by W.Tapia with DNA extracted by C. Mariani. The bone was subsampled by E. Jensen, who also extracted the DNA. DNA for both samples were converted to sequencing libraries by staff at the Yale Center for Genomic Analysis, and sequenced there.
Timing and spatial scale	NA
Data exclusions	NA
Reproducibility	NA
Randomization	NA
Blinding	NA

Did the study involve field work? ☐ Yes ☒ No

Reporting for specific materials, systems and methods

We require information from authors about some types of materials, experimental systems and methods used in many studies. Here, indicate whether each material, system or method listed is relevant to your study. If you are not sure if a list item applies to your research, read the appropriate section before selecting a response.

Materials & experimental systems

n/a	Involved in the study
☒	☐ Antibodies
☒	☐ Eukaryotic cell lines
☒	☐ Palaeontology and archaeology
☐	☒ Animals and other organisms
☒	☐ Human research participants
☒	☐ Clinical data
☒	☐ Dual use research of concern

Methods

n/a	Involved in the study
☒	☐ ChIP-seq
☒	☐ Flow cytometry
☒	☐ MRI-based neuroimaging

Animals and other organisms

Policy information about studies involving animals; ARRIVE guidelines recommended for reporting animal research

Laboratory animals	*For laboratory animals, report species, strain, sex and age OR state that the study did not involve laboratory animals.*
Wild animals	Fernanda, adult female Galapagos giant tortoise from Fernandina Island
Field-collected samples	Blood was mixed with 3 mL of Longmire Lysis buffer 40 and stored at 4° C until DNA was extracted.
Ethics oversight	The research was approved by Yale's Institutional Animal Care and Use Committee (2020-20346). The blood sample was collected under the permit MAE-DNB-CM-2016-0060-M-0003 from the Ecuador Ministry of the Environment, and imported under CITES permit 20US209142/9.

Note that full information on the approval of the study protocol must also be provided in the manuscript.
